# CIGB-300 Anticancer Peptide Differentially Interacts with CK2 Subunits and Regulates Specific Signaling Mediators in a Highly Sensitive Large Cell Lung Carcinoma Cell Model

**DOI:** 10.3390/biomedicines11010043

**Published:** 2022-12-25

**Authors:** George V. Pérez, Mauro Rosales, Ailyn C. Ramón, Arielis Rodríguez-Ulloa, Vladimir Besada, Luis J. González, Daylen Aguilar, Dania Vázquez-Blomquist, Viviana Falcón, Evelin Caballero, Paulo C. Carvalho, Rodrigo Soares Caldeira, Ke Yang, Yasser Perera, Silvio E. Perea

**Affiliations:** 1 Molecular Oncology Group, Department of Pharmaceuticals, Biomedical Research Division, Center for Genetic Engineering & Biotechnology (CIGB), Havana 10600, Cuba; 2 Department of Animal and Human Biology, Faculty of Biology, University of Havana (UH), Havana 10400, Cuba; 3 Mass Spectrometry Laboratory, Proteomics Group, Department of System Biology, Biomedical Research Division, CIGB, Havana 10600, Cuba; 4 Pharmacogenomic Group, Department of System Biology, Biomedical Research Division, CIGB, Havana 10600, Cuba; 5 Microscopy Laboratory, Department of System Biology, Biomedical Research Division, CIGB, Havana 10600, Cuba; 6 Laboratory for Structural and Computational Proteomics, Carlos Chagas Institute, Fiocruz-Paraná, R. Professor Algacyr Munhoz Mader 3775, Curitiba 81310-020, PR, Brazil; 7 Mass Spectrometry Facility, Carlos Chagas Institute, Fiocruz-Paraná, R. Professor Algacyr Munhoz Mader 3775, Curitiba 81310-020, PR, Brazil; 8 China-Cuba Biotechnology Joint Innovation Center (CCBJIC), Yongzhou Zhong Gu Biotechnology, Yongzhou 425000, China

**Keywords:** lung cancer, LCLC, CK2, CIGB-300

## Abstract

Large cell lung carcinoma (LCLC) is one form of NSCLC that spreads more aggressively than some other forms, and it represents an unmet medical need. Here, we investigated for the first time the effect of the anti-CK2 CIGB-300 peptide in NCI-H460 cells as an LCLC model. NCI-H460 cells were highly sensitive toward CIGB-300 cytotoxicity, reaching a peak of apoptosis at 6 h. Moreover, CIGB-300 slightly impaired the cell cycle of NCI-H460 cells. The CIGB-300 interactomics profile revealed in more than 300 proteins that many of them participated in biological processes relevant in cancer. Interrogation of the CK2 subunits targeting by CIGB-300 indicated the higher binding of the peptide to the CK2α′ catalytic subunit by in vivo pull-down assays plus immunoblotting analysis and confocal microscopy. The down-regulation of both phosphorylation and protein levels of the ribonuclear protein S6 (RPS6) was observed 48 h post treatment. Altogether, we have found that NCI-H460 cells are the most CIGB-300-sensitive solid tumor cell line described so far, and also, the findings we provide here uncover novel features linked to CK2 targeting by the CIGB-300 anticancer peptide.

## 1. Introduction

Nowadays, lung cancer remains the second most commonly diagnosed cancer and the leading cause of cancer-associated death worldwide [1,2]. Over the last decade, advances in research have accomplished major breakthroughs in the understanding, diagnosis and management of this disease, and now it is accepted as a heterogenous entity that can be classified into two major histologic types: small cell lung cancer (SCLC) and non-small cell lung cancer (NSCLC) [3]. Of note, NSCLC encompasses around 80–85% of lung cancer cases [4,5], and at the same time, depending on the tissue of origin, it is categorized into three main histologic subtypes: adenocarcinoma; squamous cell carcinoma; and large cell carcinoma (LCLC) [6]. Histological diagnosis of NSCLC is essential to accomplish an accurate treatment because the effectiveness of some therapeutic agents differ due to tumoral histology [4,7,8]. However, LCLC is one form of NSCLC that tends to grow more quickly and spread more aggressively than some other forms [9,10]. Furthermore, recent studies ratify that molecular screening must be taking into account in order to predict sensitivity for standard therapy [3]. Although cytotoxic chemotherapy remains an important component of systemic therapy for most patients, the majority can develop resistance or discontinue treatment as a consequence of accentuated side effects inherent to this therapy [11]. Moreover, considering that scenario, immunotherapy and molecular target therapy represent about 50% of patients with advanced NSCLC and can be applied independently or in combination with first-line therapy, contributing to the customized clinical management of patients [1]. Furthermore, up to 60% of lung adenocarcinoma and up to 50–80% of squamous cell carcinoma have a known targetable oncogenic driver mutation related with uncontrolled proliferation and cell survival [3,12]. However, the considerable percentage of patients, including those diagnosed with LCLC, remain an unmet medical need, and therefore the validation of new molecular targets is the main goal of many research groups [10,13].

Recently, protein casein kinase CK2, formerly known as casein kinase 2, has been proposed as an attractive target in cancer due to the pleiotropic roles of this kinase in cancer cells [14]. This enzyme modulates the phosphorylation of more than 300 substrates, where many of them are linked to some cancer hallmarks [2]. Structural studies have revealed that CK2 is a tetramer consisting of two catalytic (α α, α′α′ or α α′) subunits and two regulatory (β β) subunits [14]. Specifically in NSCLC, the abnormal overexpression of CK2α and CK2α′ subunits is a common feature in comparison with adjacent noncancerous tissues and correlates with poor prognosis [15,16]. On the other hand, CK2 belongs to the array of human druggable molecules and, as a result, CK2 inhibitors have an antineoplastic effect on cancer cells and its down-regulation enhances the response to standard therapy in in vivo models [2]. The CK2 inhibitors CX-4945 and CIGB-300 are the only ones tested in clinical trials where safety and antitumor activity were documented [11]. Additionally, CIGB-300 has been shown to reduce proteins linked to cisplatin resistance [17], adhesion, migration and invasion of lung cancer cells [18]. Likewise, CIGB-300 treatment did limit the vascular response in vivo using a lung cancer-induced angiogenesis model [18]. Considering the above-mentioned results, here we aimed to investigate the CIGB-300 effect on NCI-H460 cells as a model of LCLC cells. Additionally, we wanted to explore whether previous features of the CK2 pharmacological blockade elicited by CIGB-300 take place with this particular histological background.

## 2. Materials and Methods

### 2.1. Cell Culture

NCI-H460 was originally obtained from the American Type Culture Collection (ATCC, Manassas, VA, USA). The cell line was cultured in Dulbecco’s modified Eagle’s medium (DMEM) (Gibco, Waltham, MA, USA) supplemented with 10% (*v*/*v*) fetal bovine serum (FBS, Invitrogen, Carlsbad, CA, USA) and 50 µg/mL gentamicin (Sigma, St. Louis, MO, USA). Cells were maintained at 37 °C and 5% CO_2_.

### 2.2. Antiproliferative Assay

Antiproliferative effect was determined using crystal violet staining. Cells were seeded in flat-bottom 96-well plates (6000 cells/mL), and 24 h later, serial dilutions 1:2 ranging from 200 to 12.5 µM of CIGB-300 and serial dilutions 1:2 ranging from 50 to 3.12 µM of CX-4945 were added, respectively. After 48 h of incubation, the medium was removed from each well and crystal violet (1%) (Sigma, St. Louis, MO, USA) was added and incubated for 5 min at room temperature. After dye removal, wells were washed with water. Absorbance was measured in a CLARIOstar microplate reader (BMG LABTECH, Ortenberg, Germany), and half-inhibitory concentration (IC_50_) values were estimated using CalcuSyn software (v2.1) (Biosoft, Cambridge, UK).

### 2.3. Annexin V/PI Staining

Apoptosis of NCI-H460 cells was measured using FITC Annexin V Apoptosis Detection Kit (BD Biosciences, San Jose, CA, USA). Briefly, cells were incubated with 30 µM CIGB- 300 for 0.5 h, 1 h, 3 h, 6 h, 24 h and 48 h. NCI-H460 cells were incubated also with 5 µM CX-4945 for 6 h, 24 h and 48 h. Subsequently, cells were washed twice with cold PBS and resuspended in binding buffer (1X) at a final concentration of 1 × 10^6^ cells/mL. Finally, 5 µL of FITC Annexin V and PI were added, and cell suspensions were incubated for 15 min at room temperature in the dark. Flow cytometric analysis of stained cells was performed as previously described. All experiments were performed at least two times.

### 2.4. Cell Cycle Analysis

For cell cycle analysis, NCI-H460 cells were incubated with 30 µM CIGB-300 or with 5 µM CX-4945, respectively, during 6 h, 24 h and 48 h. Following treatment with the peptide, cells were collected by centrifugation, washed with PBS, and fixed at 4 °C for 2 h with ice-cold 70% ethanol. After fixation, cells were treated with DNase-free RNase A (Sigma, St. Louis, MO, USA) and subsequently stained at 37 °C for 20 min with 50 mg/mL PI solution (Sigma, St. Louis, MO, USA). Stained cells were analyzed in the above-mentioned Partec CyFlow Space flow cytometry, and FlowJo Software (v7.6.1) was used for data processing and visualization. All experiments were performed at least four times.

### 2.5. Peptide Internalization

The internalization of CIGB-300 in NCI-H460 cells was studied at 5 min, 10 min, 0.5 h, 1 h, 3 h, 6 h, 24 h and 48 h after addition of 30 µM of the peptide with N-terminal fluorescein tag (CIGB-300-F). Once incubated with the peptide, cells were washed and resuspended in PBS, and a flow cytometric analysis of cells was performed in Partec CyFlow Space instrument (Sysmex Partec GmbH, Gorlitz, Germany), and FlowJo software (v7.6.1) (BD, Ashland, OR, USA) was used for data analysis and visualization.

### 2.6. Pull-Down Experiments

For in vivo pull-down assays, N-terminal biotin tagged CIGB-300 (CIGB-300-B) was added to NCI-H460 cells at a final concentration of 30 µM and incubated for 30 min at 37 °C and 5% CO_2_. Subsequently, cells were collected, washed, and lysed in PBS solution (Sigma, St. Louis, MO, USA) containing 1 mM DTT (Sigma, St. Louis, MO, USA), Triton X-100 (1%), and complete protease inhibitor (Roche, Basel, Switzerland). Cellular lysates were cleared by centrifugation and added to 30 μL of pre-equilibrated streptavidin–sepharose matrix (GE Healthcare, Chicago, IL, USA). In the case of RNA pre-treatment, another cell extract was incubated with RNAase A (10 mg/mL) (Promega, Madison, WI, USA) for 20 min at 37 °C. Following 1 h at 4 °C, the matrix was collected by centrifugation and extensively washed with cold PBS 1 mM and DTT. Proteins bound to streptavidin–sepharose matrix were digested with trypsin (Promega, Madison, WI, USA) during 16 h or eluted for Western blot analysis. In parallel, untreated cells were subjected to the same experimental procedure to identify those proteins non-specifically bound to streptavidin–sepharose matrix. Proteins were resolved in 12% SDS-PAGE and then transferred to a nitrocellulose membrane. For Western blot analysis, a mouse monoclonal antibody against CK2α (Abcam, Cambridge, UK), a rabbit polyclonal antibody against CK2α′ (Abcam, Cambridge, UK), a mouse monoclonal antibody against CK2β (Sigma, St. Louis, MO, USA), and monoclonal rabbit antibodies against PTEN and RPS6 (Cell Signaling, Danvers, MA, USA), respectively, were used as primary antibodies. Detection was performed with peroxidase-conjugated anti-mouse IgG 1:5000 (Sigma, St. Louis, MO, USA) and anti-rabbit IgG 1:5000 (Sigma, St. Louis, MO, USA). In parallel, untreated cells were subjected to the same experimental procedure to identify those proteins non-specifically bound to streptavidin–sepharose matrix. Detection was performed with peroxidase conjugated anti-mouse or anti-rabbit IgG (Sigma, St. Louis, MO, USA), and a signal was developed using SuperSignal West Pico Chemiluminescent Substrate (Thermo Fisher Scientific, Waltham, MA, USA). All experiments were performed at least two times.

### 2.7. LC-MS/MS Analysis and Protein Identification

The samples were analyzed at the mass spectrometry facility RPT02H/Carlos Chagas Institute–Fiocruz Paraná. Each peptide mixture fraction was suspended in 0.1% formic acid and analyzed three times as follows. The setup used a nanoLC-1D plus (Sciex, Framingham, MA, USA) coupled online with an LTQ-Orbitrap XL mass spectrometer (Thermo Fisher Scientific, Waltham, MA, USA). The peptide mixture was loaded in a 15 cm in length with a 75 μm I.D.) packed in-house with ReproSil-Pur C18-AQ 3 μm resin (Dr Maisch GmbH HPLC) for peptide separation. The flow rate was 250 nl/min, and we applied a 60 min gradient, first using 95% of mobile phase A (0.1% formic acid, 5% DMSO) and then 40% of mobile phase B (0.1% formic acid, 5% DMSO in acetonitrile). The effluent from the nLC column was directly electrosprayed into the mass spectrometer. The instrument was set to data-dependent acquisition to automatically switch between full scan MS and MS/MS acquisition with a dynamic exclusion of 90 s. Survey scans (300–1800 *m*/*z*) were acquired in the Orbitrap system with a resolution of 60,000 at *m*/*z* 400 (AGC target ions accumulation of 1,000,000). The 10 most-intense ions (70–2000 *m*/*z*) with charge states of +2 or +3 were sequentially isolated and fragmented in the higher-energy collisional dissociation (HCD) collision cell using a normalized collision energy of 35. The fragment ions were analyzed with a resolution of 17,500 at 200 *m*/*z*. The general mass spectrometric conditions were as follows: 2.70 kV spray voltage, 100 μA source current, no sheath and auxiliary gas flow, heated capillary temperature of 175 °C, predictive automatic gain control (AGC) enabled.

Identification of peptides and proteins was based on the match between-runs procedure using MaxQuant software (v1.6.2.10) (Cox et al., 2014), considering oxidation (M), deamidation (NQ), and N-terminal acetylation as variable modifications and propionamide cysteine as fixed modification. For the alignment of the chromatographic runs, default parameters (20 min alignment time window and matching of 0.7 min between runs) were used. UniprotKB Human database was used for 1% false-discovery-rate protein identification. Filtering and relative quantification between CIGB300-treated and non-treated were conducted with Perseus computational platform (v1.6.13.0) (Tyanova et al., 2016). After filtering for three valid replicate values in at least one group, a Student’s T two-sample test was employed to identify statistically significant changes (*p*-values lower than 0.05) in protein levels. Proteins increasing 1.5-fold over the non-treated group were selected.

### 2.8. Confocal Microscopy

NCI-H460 cells were plated on eight-well glass slides and incubated overnight at 37 °C and 5% CO_2_. After incubation, cells were treated with 30 μM biotinylated CIGB-300. Subsequently, cells were washed with cold PBS three times and fixed in 10% formalin for 10 min at 4 °C. After permeabilization with 0.2% Triton X-100 for 10 min, cells were blocked by incubation with 4% bovine serum albumin (Sigma, St. Louis, MO, USA) in PBS for 30 min at room temperature and then washed. Proteins were detected by employing antibodies previously described at 1:200 for 2 h at room temperature. Finally, anti-rabbit or anti-mouse IgG Alexa Fluor 594 conjugate at 1:250 dilution (Cell Signaling Technology, Danvers, MA, USA) and Streptavidin-FITC at 1:2000 (Sigma, St. Louis, MO, USA) were incubated for 1 h at room temperature and washed three times with PBS. Coverglasses were mounted using Vectashield mounting medium with DAPI (Vector Laboratories, Burlingame, CA, USA) and analyzed using an Olympus FV1000 confocal laser scanning microscopeIX81 laser scanning fluorescence microscope (Olympus, Tokyo, Japan). Images were acquired with UPLSAPO 40X immersion objective and processed using Olympus FluoView software (v4.0) (Olympus, Tokyo, Japan). Five optical fields or Z-stacks were examined for each experimental condition.

### 2.9. CK2 Subunits and Downstream Signaling

NCI-H460 cells were plated on six-well cell culture plates at 1 × 10^6^ cells per well and incubated overnight at 37 °C and 5% CO_2_. Then, cells were incubated with 30 µM CIGB-300 for 0.5 h, 1 h, 3 h, 6 h, 24 h and 48 h and with 5 µM CX-4945, respectively, for 6 h, 24 h and 48 h. After incubation, the culture medium was withdrawn, and cells were washed with PBS and lysed in RIPA buffer containing protease/phosphatase inhibitor (Thermo Fisher Scientific, Waltham, MA, USA). Western blot detection was carried out as described (4.6). Primary antibodies against phospho-PTEN (S380) and total PTEN (Cell Signaling Technology, Danvers, MA, USA), phospho-RPS6 (S235/236) and total RPS6 (Cell Signaling Technology, Danvers, MA, USA), Ezrin (Abcam, Cambridge, UK), c-Myc (CIGB, SP, Cuba), and Fibrillarin (Abcam, Cambridge, UK) were used. Primary antibodies against CK2 subunits were used as previously described. All experiments were performed at least two times.

### 2.10. Quantitative Real-Time PCR Assays

NCI-H460 cells were plated on six-well cell culture plates at 1 × 10^6^ cells per well and incubated overnight at 37 °C and 5% CO_2_. Briefly, cells were incubated with 30 µM CIGB-300 for 0.5 h, 3 h and 6 h and with 5 µM CX-4945, respectively, for 3 h, 6 h, 16 h and 24 h. Three replicates per condition were used. After incubation time, the culture medium was withdrawn, and the cells were washed with PBS and suspended in 350 μL of lysis buffer (with 1% of β-mercaptoethanol, Sigma, St. Louis, MO, USA) for RNA isolation (AllPrep DNA/RNA/miRNA Universal Kit, Qiagen, Valencia, CA, USA), according to the manufacturer protocol. All RNA samples were checked by Nanodrop spectrophotometer to measure concentration (ng/μL) and OD relation (260/280 nm). Quality control parameters were fulfilled by all the samples (100% OD 260/280 nm between 1.7 and 2.2). Complementary (c)DNAs were obtained from 500 ng of total RNAs, using the Transcriptor First Strand cDNA Synthesis Kit package (Roche, Mannheim, Germany), following manufacturer instructions. The qPCR reactions were set up in 20 μL using LightCycler^®^ 480 SYBR Green I Master 2x (Roche, Mannheim, Germany), 300 nM of oligonucleotides, and 1:10 dilutions of each cDNA, with three replicates per sample. We amplified two genes for normalization of GAPDH (glyceraldehyde-3-phosphate dehydrogenase) and HMBS (hydroxymethyl-bilane synthase), and the transcripts encoding for CK2 subunits. All the oligonucleotides were synthetized in the Synthesis Department at CIGB (Table S1). Runs were carried out in the LightCycler^®^ 480II equipment (Roche, Mannheim, Germany) in a 96-well format and SYBR Green Probe II mode with a standard program with 45 cycles. Fold changes for transcripts in each treatment were calculated with respect to time-matched control groups after normalization with GAPDH and HMBS genes (v2.0.13, Qiagen GbmH, Munich, Germany), using Ct values and reaction efficiencies per amplicon calculated in LinReg 2009 (v11.3). Statistical differences are reported in this program, with a *p*-value-associated with a significance for *p* < 0.05.

### 2.11. Statistical Analysis

Differences between groups were determined using one-way ANOVA followed by Tukey’s multiple comparisons test. Data were analyzed and represented in GraphPad Prism (v6.01) (GraphPad Software, Inc., San Diego, CA, USA).

## 3. Results

### 3.1. NCI-H460 Is Highly Sensitive to CIGB-300 Peptide

In order to evaluate CK2-mediated inhibition using CIGB-300 in an NCI-H460 cell line, we performed an antiproliferative assay using the Crystal Violet approach, followed by apoptosis and cell cycle assays using flow cytometry analysis.

**Figure 1:**
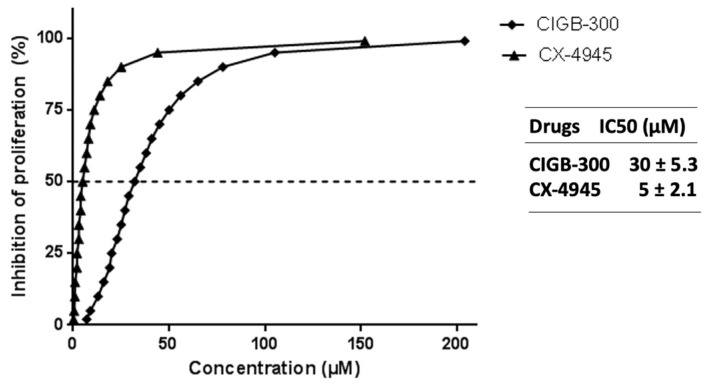
Crystal Violet assay in NCI-H460 cell line. Dose-response curve corresponding to the antiproliferative effect of CIGB-300 and CX-4945. Values are shown as mean, *n* = 4, R = 0.98.

As a result, CIGB-300 and CX-4945 strongly inhibited the proliferation of NCI-H460, where a dose-response pattern was noticeable, reaching IC_50_ values of IC_50_ = 30 ± 5.3 µM and IC_50_ = 5 ± 2.1 µM, respectively (Figure 1). Of note, these LCLC cells are the cell line most sensitive toward the antiproliferative effect of CIGB-300, compared to other solid tumor cells whose IC_50_ mean value is about 60–300 µM [19].

**Figure 2:**
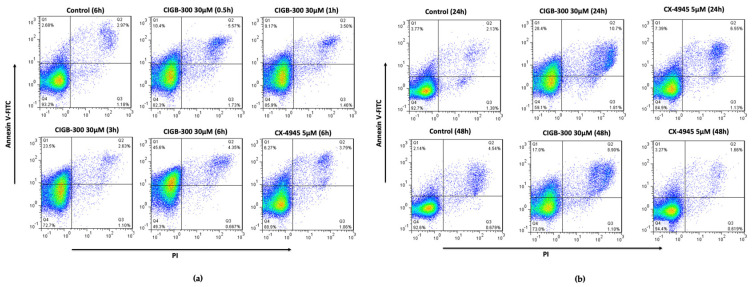
Apoptotic profile of CIGB-300 and CX-4945 in NCI-H460 cell line at IC_50_ values using a double staining with Annexin V-FITC and PI: (**a**) Short incubation times; (**b**) Later incubation times.

CIGB-300 treatment affected the NCI-H460 cell viability in a time-course manner, with the apoptotic peak at 6 h (near 50%) (Figure 2a). A slight recovery of the cell population 24 h and 48 h post treatment was observed (Figure 2b). Of note, CX-4945 did not induce any remarkable changes in the double-positive region of the panel (Figure 2b). On the other hand, slight perturbations (10–15%) of cells accumulated in the G0/G1 and S phases in the cell cycle of NCI-H460 cells were observed after CK2 inhibition by CIGB-300 (Figure S1). Otherwise, CX-4945 treatment modified the amount of the cell population in all phases of the cell cycle in comparison with the untreated cells (Figure S1).

### 3.2. CIGB-300 Internalization and Interaction Profile in NCI-H460 Cells

**Figure 3:**
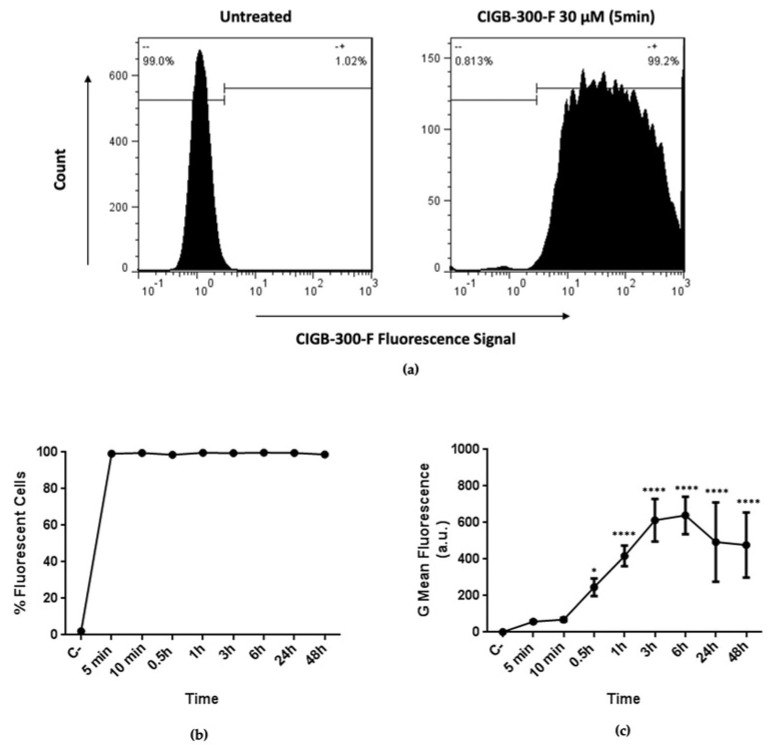
Internalization kinetics of CIGB-300-F in NCI-H460 cell line: (**a**) Fluorescent signal of CIGB-300-F at 30 µM in NCI-H460 cells after 5 min of incubation vs. untreated cells; (**b**) Percent of fluorescent cells after different times of incubation with 30 µM of CIGB-300-F; (**c**) Kinetics of intracellular accumulation of CIGB-300-F at 30 µM. Viable cells were gated using FSC vs. SSC dot plot. Autofluorescence of untreated cells were set as threshold. Statistical comparisons were performed between each treated conditions vs. the untreated cells. Values from (**c**) are shown as mean ± SD, *n* = 3. (*) *p*-value < 0.05, (****) *p*-value < 0.0001.

To better understand the CIGB-300 effect in NCI-H460 cells, we studied the peptide internalization using N-terminal fluorescein tagged peptide (CIGB-300-F). We found that CIGB-300-F rapidly internalized into NCI-H460 cells, reaching virtually the whole cell population after only 5 min of incubation (Figure 3a). Importantly, the percentage of fluorescent cells remained unchanged for longer incubation times, including 24 and 48 h (Figure 3b). Conversely, the intracellular accumulation of the peptide exhibited a different kinetic, with a slight saturation between 3 and 6 h of incubation, followed by a decrease of fluorescent intensity for later times (Figure 3c).

**Figure 4:**
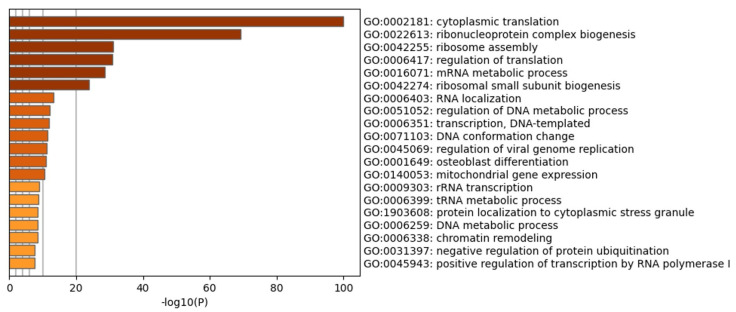
Enrichment analysis of CIGB-300 interacting proteins in NCI-H460 cells. Biological processes significantly represented in the peptide interaction profile (*p*-value < 0.01, enrichment factor > 1.5), were identified using Metascape gene annotation and analysis resource (https://metascape.org/, accessed on 1 September 2022).

Once we demonstrated the fast and efficient internalization of CIGB-300, its interaction profile was explored using N-terminal biotin tagged peptide (CIGB-300-B). In vivo pull-down experiments followed by LC-MS/MS analysis allowed us to identify a group of 322 proteins conforming the CIGB-300 interactome in NCI-H460 cells (Table S1). Functional analysis evidenced that the identified proteins are mainly involved in nucleic acid metabolic processes (including transcription and translation), DNA conformation and chromatin remodeling, ribosome biogenesis/assembly, as well as protein localization and degradation through ubiquitination (Figure 4). In agreement with functional enrichment, network analysis revealed that protein complexes related to transcription, translation, RNA processing and splicing, chromosome organization, and regulation of innate immune response appeared to be represented in the CIGB-300 interactome (Figure S2).

Considering that the CIGB-300 peptide was originally conceived to block protein kinase CK2 phosphorylation through interaction with the phosphoacceptor domain, we searched for CK2 substrates among the peptide interacting proteins in NCI-H460 cells. Of note, a total of 29 well-documented CK2 substrates, including CK2 alpha subunit (CSNK2A1) and nucleophosmin 1 (NPM1), were identified in the CIGB-300 interactome (Table 1). CK2 substrates such as histone H4 (HIST1H4A), histone deacetylase 2 (HDAC2), members of the heterogeneous nuclear ribonucleoprotein family (HNRNPA1, HNRNPA2B1, HNRNPC), DNA topoisomerases 1 and 2 alpha (TOP1, TOP2A), eukaryotic translation initiation factor 2 subunit 2 (EIF2S2), Ras GTPase-activating protein-binding protein 1 (G3BP1), and others with critical roles in cancer cell physiology were also identified.

### 3.3. CIGB-300 Differentially Interacts with CK2 Subunits

We have previously documented a dual mechanism of CIGB-300’s inhibition of the CK2-mediated phosphorylation in cancer cells (i.e., involving either substrates and/or CK2α subunits) [20]. Moreover, although CIGB-300-CK2α interaction was detected, we do not discard the possibility that CIGB-300 interaction with CK2 subunits can be molecularly more complex than the mass spectrometry approach revealed. Thus, we aimed to explore the putative interaction of CIGB-300 with each CK2 subunit and selected CK2 substrate/non-substrate proteins from the AKT signaling pathway in a highly sensitive cellular background such as the NCI-H460 model. For those purposes, in vivo pull-down followed by Western blot and in situ confocal microscopy assays were carried out.

**Figure 5:**
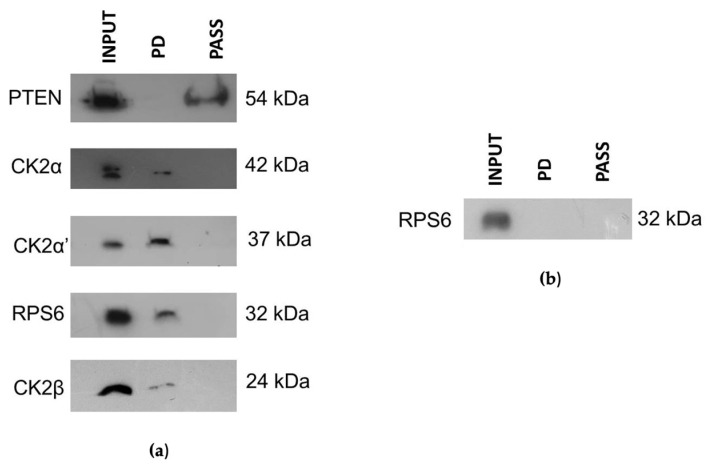
Pull-down in vivo assay. CIGB-300-B was added to NCI-H460 cells at a final concentration of 30 µM and incubated for 0.5 h: (**a**) RNAase free assay; (**b**) RNAase assay for RPS6. INPUT: Cell extract. PD: Fraction interacting in pull-down reaction. PASS: Fraction not interacting in pull-down reaction.

CIGB-300 mainly interacted with the CK2 α and α′ catalytic subunits, while only minor levels of the regulatory subunit (β) of CK2 were observed in the pull-down fraction (Figure 5a). Interestingly, CIGB-300 binding seemed more evident with CK2α′ than CK2α. Otherwise, CIGB-300 did not interact with PTEN, which is a direct CK2 substrate (Figure 5a). However, the signal of RPS6 protein in the pull-down fraction indicated an evident interaction with CIGB-300 (Figure 5a), but rather, that interaction seemed to be facilitated by RNA instead of the protein–protein complex (Figure 5b).

**Figure 6:**
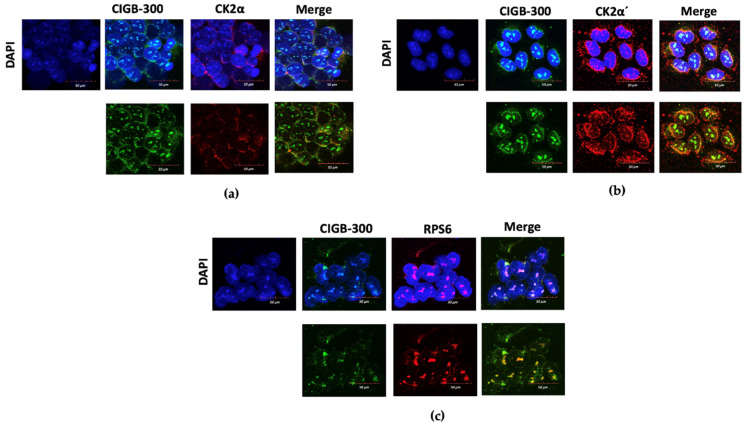
Confocal microscopy in NCI-H460 cells: (**a**) Interaction panel of CIGB-300 and CK2α; (**b**) Interaction panel of CIGB-300 and CK2α′; (**c**) Interaction panel of CIGB-300 and RPS6. Blue fluorescence: nuclear DAPI stain. Green fluorescence: CIGB-300-B was incubated at IC_50_ value during 0.5 h and identified with Streptavidin-FITC. Red fluorescence: Corresponding proteins were identified with a primary antibody and then with a secondary antibody conjugated to AlexaFluor 500. Orange fluorescence: Merge of Green/Red channels representing colocalization signals. Scale bars: 50 µm.

To additionally support the in vivo pulldown data, we looked for the in situ physical proximity of CIGB-300 with the above-mentioned proteins by confocal microscopy. Data from microscopy panels showed the well-defined location of CIGB-300 in cytosol and the perinuclear and nucleolus compartment of NCI-H460 cells. The peptide mainly remained in the nucleolus of cells (Figure 6). In the case of CK2 subunits, CK2α and CK2β showed a preferential location in cytosol and in the perinuclear compartment of cells, as previously reported (Figure 6a and Figure S3) [21], whereas CK2α′ was also located in the cell nucleolus, similar to CIGB-300 (Figure 6b). Overall, the main interactions between CIGB-300 and each CK2 subunit (Merge panel of Figure 6a,b and Figure S3) were in cytosol and in a minor proportion in the perinuclear compartment of cells. The interaction of CIGB-300 with RPS6 protein was at the nucleolus, where this protein was mainly localized (Figure 6c).

### 3.4. CIGB-300 Regulates Some CK2 Downstream Signaling Mediators in NCI-H460 Cells

**Figure 7:**
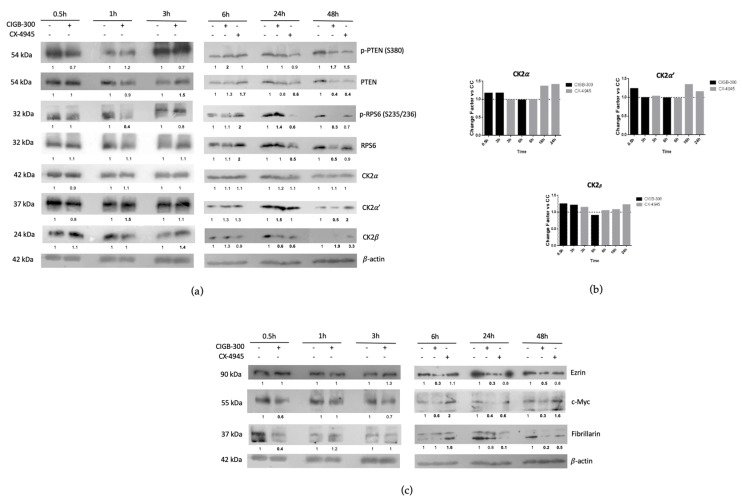
Time-course analysis of treatment with CIGB-300 and CX-4945 in NCI-H460: (**a**) Regulation of CK2 subunits and CK2 substrate/non-substrate proteins by Western blot; (**b**) mRNA levels of CK2 subunits; (**c**) Regulation of some cancer molecular markers after CK2 inhibition by Western blot. Expression levels were relativized with β-actin protein and appropriated housekeeping genes, respectively. Each treatment was also compared with its untreated condition.

To explore the regulation of CK2 levels by CIGB-300 treatment, we firstly performed time-course experiments focused on the regulation of each CK2 subunit by Western blot and qPCR assays. Furthermore, we analyzed the levels of PTEN, RPS6 and other proteins related to CK2 signaling in NCI-H460 cells. Importantly, data from Figure 7a show that CIGB-300 and CX-4945 treatment altered the CK2α′ and CK2β expression levels at longer incubation times, and no evident fluctuations in the CK2α catalytic subunit were observed. Interestingly, mRNA levels of CK2 subunits seemed to show a discrete up-regulation (no more than 1.5-fold) at earlier times with CIGB-300 treatment and longer incubation times for CX-4945 treatment (Figure 7b). Likewise, data from time-course experiments indicated that relevant inhibitory effects of p-PTEN (S380)/PTEN and p-RPS6 (S235/236)/RPS6 were observed from 6 h to 48 h post treatment. Otherwise, the regulation of other molecular cancer markers was more noticeable at longer incubation times of treatment (Figure 7c), except in the case of c-Myc and Fibrillarin proteins, where a reduction at earlier times after CIGB-300 incubation is also evident.

## 4. Discussion

Lung cancer constitutes a major public health problem worldwide, and it is the most common cause of cancer-related death in men and the second most common in women [7]. Additionally, target therapy is considered the cornerstone of NSCLC management and has significantly improved patient outcomes and quality of life [22]. Therefore, the development of novel molecular targets is a primary goal in NSCLC cancer therapy [23,24]. Numerous pieces of evidence suggest that casein kinase 2, or CK2, could be considered an attractive target for target therapy [20,25]. Furthermore, the overexpression of any of the catalytic subunits of CK2 (α, α′) in NSCLC correlates with unfavorable prognosis and overall survival [26]. Moreover, CK2 inhibition in NSCLC is related to apoptosis [27]; cell cycle arrest [28]; decrease of cell adhesion, migration and invasion [17]; limitation of vascular response to angiogenesis [18]; synergy with radiation and chemotherapeutic agents [16,29]; and sensitization of drug-resistant cells [11,25].

In this work, we evaluated for the first time the molecular events supporting CK2 inhibition by CIGB-300 in an LCLC in vitro model such as NCI-H460 cells. Contrary to what we have previously found in solid tumor cells, CIGB-300 displayed a very low IC_50_ value (30 µM), which is indicative that NCI-H460 is a tumor cell line highly sensitive to this peptide inhibitor. In fact, CIGB-300 showed higher IC_50_ values in other NSCLC cell lines such as NCI-H125 (IC_50_ = 60 µM) [19] and A549 (IC_50_ = 171 µM) [30]. Interestingly, the IC_50_ value for CIGB-300 on NCI-H460 cells was quite similar to those observed for different kinds of blood cancer cells, with values of half inhibitory doses ranging from 20 to 35 µM [20]. Whether the putative CIGB-300-relevant targets on LCLC cells are closer to those on blood cancer cell lines than those on other NSCLC cells, it remains to be determined. The CX-4945 used in this work as a reference of CK2 inhibition exhibited an IC_50_ = 5 µM as previously reported by other groups [25], however this does not represent a head-to-head comparison with CIGB-300 because of the distinct chemical nature of both compounds.

Taking into account the apoptotic effect broadly described for CIGB-300 in cancer cell lines [19,20,31], we also performed here a double stain using Annexin V-FITC/PI in a flow cytometry approach as part of the characterization of the high sensitivity of these LCLC cells toward CIGB-300. As expected, CIGB-300 compromised cell viability at early times [19], and the major induction of apoptotic cells was at 6 h post treatment, which corresponds with maximum value of the G mean of fluorescence reported in the internalization assay. Similar results were obtained by Perera et al., 2012 for other cell lines, although they also correlated cell sensitivity with the subcellular localization of CIGB-300 in solid tumor cell lines [19]. Nevertheless, apoptotic percentages at 24 h and 48 h post treatments were lower in comparison with 6 h. This might be attributable to the high proliferation rate of NCI-H460 cells (17.8 h) (www.dtp.cancer.gov, accessed 3 August 2022) and putative CIGB-300 peptide degradation at longer incubation times [30]. Of note, CX-4945 inhibitor had a mild effect on apoptosis despite the NCI-H460 cell line showing high sensitivity to this CK2 inhibitor. CK2 is a well-documented regulator of the cell cycle [26], thus we assessed a flow cytometry assay on NCI-H460 cells treated with CK2 inhibitors to verify its distribution. However, CIGB-300 only displayed a slight accumulation at around 10–15% of cells in S phase at 6 h, and in G0/G1 at later times of incubation (24 h and 48 h). In the case of CX-4945, modifications in the cell cycle profile were heterogeneous during the assay; some authors have noted similar results according to the cancer cell niche and sensitivity to this compound [32,33,34]. Overall, the extent of cell cycle impairment by CIGB-300 and CX-4945 indicate that such a “cytostatic” effect does not contribute significantly to the observed antiproliferative outcomes in NCI-H460 cells.

Once we explored the sensitivity of these LCLC cells towards the antiproliferative effect of CIGB-300, we next characterized the internalization kinetics using the fluorescent CIGB-300 peptide (CIGB-300-F). Data evidenced that this peptide quickly internalized to NCI-H460 cells and reached almost all the cell population interrogated by flow cytometry. However, this internalization profile resembles that previously reported for CIGB-300 by Benavent et al., 2014 and Rosales et al., 2021, and it is similar to that described for others using Tat peptides [20,30]. Likewise, data from the temporal intracellular accumulation of CIGB-300 showed that peptide internalizes into NCI-H460 cells in a time-dependent manner until 3 h post treatment, with a subsequent slight plateau at 6 h, which could be related with a saturation of internalization despite CIGB-300 using different pathways of entrance in lung cancer cells [30].

The CIGB-300 interaction profile was also explored in NCI-H460 cells using pull-down experiments followed by LC-MS/MS analysis. In line with cellular effects attained by this peptide inhibitor in NCI-H460 and other cell models, CIGB-300 interacted with several proteins involved in proliferation, cell death and cell cycle regulation [21,31]. Furthermore, the search for CK2 substrates in the CIGB-300 interactome revealed that the peptide is able to interact with substrates playing central roles in cancer cell physiology, such as protein DEK, G3BP1, HDAC2, DNA topoisomerase 1 and 2-alpha, and others with oncogenic potential. Interestingly, the CK2α subunit was identified among CIGB-300 interacting proteins, reinforcing previous findings in lung cancer and leukemic cells that suggest a direct impairment of CK2 enzymatic activity by this peptide inhibitor [20,21,35].

CIGB-300 is a peptide selected to disrupt CK2-mediated phosphorylation by direct binding to the conserved phosphoacceptor site on their substrates [19], and considering that Perera et al., 2020 and Rosales et al., 2021 have proposed an alternative mechanism by direct interaction of the peptide with the CK2 catalytic subunit (CK2α), we performed here in vivo pull-down assays followed by Western blot [20,35]. Similar to previous findings, CIGB-300 did not bind to CK2 substrate PTEN and it bound to the RPS6 protein in vivo, which is indirectly phosphorylated by the CK2-activated S6 protein kinase [36]. Noteworthily, the interaction of CIGB-300 and RPS6 is more RNA-mediated than protein–protein dependent since RNAase pre-treatment abrogated the signal at the pull-down lane. Similar result with the C23 protein was obtained by Perera et al., 2009 in other lung cancer cell lines. In the case of CK2 subunits, CIGB-300 clearly interacted with CK2α and its isoform CK2α′, which are the catalytic subunits of this enzyme. Interestingly, the interaction with CK2α′ was more evident than that observed for CK2α, despite having the same amount of protein in the input. On the other hand, the interaction with the regulatory subunit (CK2β) was faint, and that might be the result because the CIGB-300 pulled the CK2 holoenzyme and kept some CK2β residual subunits after washing the matrix. We speculated that this apparently stronger binding of the CK2α′ could be a hallmark in CIGB-300-sensitive cancer cells; further studies need to be assessed in order to respond to that interrogative. To support the in vivo pull-down data, we looked for the in situ molecular proximity of CIGB-300 with these proteins in different subcellular compartments by using confocal microscopy. CIGB-300 colocalized with each CK2 subunit, mainly in cytosol and partly in the perinuclear compartment of NCI-H460 cells, as previously observed for CIGB-300-CK2α [35]. Similar to the in vivo pull-down data, more-evident merged colocalization was observed for CIGB-300 and CK2α′ on these LCLC cells. Taking into consideration that CIGB-300-CK2β interaction was negligible in the pull-down assay, we believed that the merge panel must be through the interaction with the CK2 holoenzyme instead of with the subunit alone. In addition, major signals of interaction between CIGB-300 and RPS6 occurred at the nucleolus of cells.

Finally, we performed Western blot and quantitative real-time PCR experiments to characterize the putative CK2 subunits’ regulation by CIGB-300 and CX-4945, and PTEN and RPS6 proteins were also included in this analysis. CK2α protein levels did not suffer any affectation throughout the time course in our experimentation. Surprisingly, CK2α′ and CK2β protein levels experienced an evident modulation after longer periods of incubations with CIGB-300 that was not preceded by an affectation of the respective mRNA levels. In the case of p-PTEN (S380)/PTEN, CIGB-300 affected phosphorylation at earlier times, and modulated protein levels at 3 h after incubation. However, at longer incubation times with CIGB-300 and CX-4945, modulation was more evident at both levels, where p-PTEN (S380) and PTEN signals notably declined in comparison with the untreated cells at 48 h. In addition, CK2-dependent phosphorylation of PTEN has double and counterintuitive effects, because it increases PTEN stability but also inhibits its lipid phosphatase activity [37]. In fact, the CX-4945 compound has demonstrated the regulation of the PI3K/AKT pathway through PTEN activity in T cell acute lymphoblastic leukemia [38]. Concerning the RPS6 protein, it was susceptible to the CIGB-300 effect at earlier incubation times; and at later times, the CX-4945 effect fluctuated with the up-regulation and down-regulation of p-RPS6 (S235/236)/RPS6 expression levels. Furthermore, CIGB-300 induced a potent inhibition of p-RPS6 (S235/236) at 48 h, where it also affected the RPS6 protein levels. RPS6 is a component of the 40S small ribosomal subunit and behaves as a ribosomal RNA-binding protein [39]. Additionally, RPS6 plays important roles in ribosome biogenesis, protein translation, cell proliferation, cell growth, DNA repair, apoptosis, cell differentiation, and glucose metabolism in both normal and cancer cells. Substantial evidence suggests that RPS6 is a potential therapeutic target, and knockdown experiments have demonstrated that RPS6 itself, not only p-RPS6, is indispensable for the proliferation or survival of various cancer cells [36]. Importantly, knockdown of CSNK2A1 (the gene encoding CK2α) or CSNK2B (the gene encoding CK2β) has been shown by others to reduce the p-RPS6 (S235/236) in an AKT/mTORC1/S6K1-dependent manner in HK-2 cells [37]. Therefore, the pharmacological targeting of CK2 catalytic subunits by CIGB-300 observed here could recapitulate the effect of genetic knockdown of such subunits on the p-RPS6 and RPS6 protein levels on these LCLC cells. Altogether, we can assume that the CK2 inhibition mechanism of PTEN by CIGB-300 is not through the blockade of the CK2 phosphosite in substrates but rather due to CK2 targeting by the peptide. However, in the case of RPS6, the inhibition mechanism must operate at several levels as it is downstream in different pathways and CIGB-300 could block S6 kinase (CK2 substrate), which is its main phosphodonor. Thus, CIGB-300 might interrupt the phosphorylation of RPS6 in a multitarget manner, resulting in a strong affectation of the biological function of this protein.

CK2 activity is primordial for plenty of signaling pathways and proteins that potentiate malignancy in tumoral cells. In this work, we finally interrogated how CIGB-300 could impact some cancer poor-prognosis markers such as Ezrin, c-Myc and Fibrillarin in an NCI-H460 cell model. Interestingly, Ezrin down-regulation was more evident only after CIGB-300 treatment at later times. This cancer-linked biomarker is overexpressed in a variety of tumoral tissues and is associated with different pathways including protein kinase C, Rho-kinase, NF-ĸB and PI3 kinase/AKT [40]. Therefore, the down-regulation elicited by CIGB-300 could contribute in part to inhibiting the well-documented invasive role of this protein in cancer cells [41]. On the other hand, c-Myc protein dysregulation is correlated with aggressive, undifferentiated tumors and poor clinical response [42]. Several studies have reported c-Myc target as a representative target for the therapeutic intervention of NSCLC [43]. Moreover, c-Myc oncoprotein has been shown to be overexpressed in about 50–75% of NSCLC cases [44], and Rapp et al., 2009 reported that this protein was a key metastatic and angiogenesis inducer in an NSCLC mouse model [45]. In our work, CX-4945 induced c-Myc up-regulation, and that result might be a consequence of the cell rescue response after CK2 inhibition with this compound. However, the CK2-mediated phosphorylation status, which can influence c-Myc dimerization, DNA binding capacity, and its oncogenic activity [42], was not interrogated in this work. Otherwise, CIGB-300 treatment decreased c-Myc protein expression throughout all incubation times, which could also contribute in part to the NCI-H460 cells’ high sensitivity towards the peptide treatment. Finally, Fibrillarin expression had a very similar modulation to p-RPS6 (S235/236) levels after CK2 inhibition with both compounds. The Fibrillarin protein is a nucleolar stress marker that catalyzes the 2′ O methylation of rRNAs to manage the translation of mRNAs [46]. Fibrillarin overexpression promotes cellular proliferation, poor prognosis, and resistance to the chemotherapy of MCF-7 breast cancer cells [34,47]. Other experiments indicate that p53 regulates this molecule, and the down-regulation of c-Myc results in a decrease in the level of Fibrillarin expression [48]. Our data could suggest that the down-regulation of c-Myc protein levels by CIGB-300 treatment could lead to a decrease of Fibrillarin levels.

All in all, we have provided here the first evidence of the impact of inhibiting CK2 on LCLC cell viability as well as a group of molecular findings that might support the high cell sensitivity of NCI-H460 cells towards CIGB-300. Whether the preferential binding of CIGB-300 to CK2α′ along with the inhibition of the cancer signaling mediators represents distinctive features only for the high CIGB-300 cell sensitivity deserves further experiments, in order to reinforce this hypothesis.

## 5. Conclusions

LCLC NCI-H460 is a unique solid tumor cell line highly sensitive to CIGB-300. Peptide internalization kinetics, apoptosis and cell cycle arrest were some features that partly justified the potent antiproliferative effect regarding CK2 inhibition. Interestingly, the unexpected differential interaction of CIGB-300 with the CK2 subunits represents not-previously documented molecular events. Additionally, the regulation of PTEN, RPS6 and other relevant cancer-linked markers by CIGB-300 treatment provides new clues about how CIGB-300 could modulate several signaling pathways in this cell model. All in all, our data suggest the notion of using the pharmacological blockade of CK2 as a promising strategy to treat LCLC.

## Figures and Tables

**Table 1:** Protein kinase CK2 substrates identified among the CIGB-300 interacting proteins in NCI-H460 cells.

Uniprot ACC	Gene Names	Protein Names
Q8NE71	*ABCF1*	ATP-binding cassette sub-family F member 1
P20290	*BTF3*	Transcription factor BTF3
Q07021	*C1QBP*	Complement component 1 Q subcomponent-binding protein, mitochondrial
P68400	*CSNK2A1*	Casein kinase II subunit alpha
P35659	*DEK*	Protein DEK
P20042	*EIF2S2*	Eukaryotic translation initiation factor 2 subunit 2
Q13283	*G3BP1*	Ras GTPase-activating protein-binding protein 1
Q92769	*HDAC2*	Histone deacetylase 2
P62805	*HIST1H4A*	Histone H4
P09651	*HNRNPA1*	Heterogeneous nuclear ribonucleoprotein A1 processed
P22626	*HNRNPA2B1*	Heterogeneous nuclear ribonucleoproteins A2/B1
P07910	*HNRNPC*	Heterogeneous nuclear ribonucleoproteins C1/C2
Q16666	*IFI16*	Gamma-interferon-inducible protein 16
P46821	*MAP1B*	Microtubule-associated protein 1B
Q9UKD2	*MRTO4*	mRNA turnover protein 4 homolog
P19338	*NCL*	Nucleolin
Q14978	*NOLC1*	Nucleolar and coiled-body phosphoprotein 1
P06748	*NPM1*	Nucleophosmin
Q13393	*PLD1*	Phospholipase D1
Q15287	*RNPS1*	RNA-binding protein with serine-rich domain 1
P46777	*RPL5*	60S ribosomal protein L5
Q96SB4	*SRPK1*	SRSF protein kinase 1
P05455	*SSB*	Lupus La protein
P43307	*SSR1*	Translocon-associated protein subunit alpha
Q08945	*SSRP1*	FACT complex subunit SSRP1
P53999	*SUB1*	Activated RNA polymerase II transcriptional coactivator p15
Q13428	*TCOF1*	Treacle protein
P11387	*TOP1*	DNA topoisomerase 1
P11388	*TOP2A*	DNA topoisomerase 2-alpha

## Data Availability

All data are already presented in the manuscript and available on request from the corresponding author.

## Supplementary Materials

The following supporting information can be downloaded at: https://www.mdpi.com/article/10.3390/biomedicines11010043/s1, Figure S1: Cell cycle modulation by CIGB-300 and CX-4945 in NCI-H460 6 h, 24 h and 48 h post treatment; Figure S2: Protein–protein interaction networks associated with CIGB-300 interacting proteins in NCI-H460 cells; Figure S3: Confocal microscopy in NCI-H460 cells of CIGB-300 and CK2β; Table S1: Interactomics profile of CIGB-300 in NCI-H460 cells.
