# Supplementary material for: CIGB-300 Anticancer Peptide Differentially Interacts with CK2 Subunits and Regulates Specific Signaling Mediators in a Highly Sensitive Large Cell Lung Carcinoma Cell Model
Source: Biomedicines. 2022 Dec 25;11(1):43. doi: 10.3390/biomedicines11010043 (PMC9856093; doi:10.3390/biomedicines11010043)
Supplement: Supplementary file 1 [file biomedicines-11-00043-s001.zip › Figure S1.pdf]

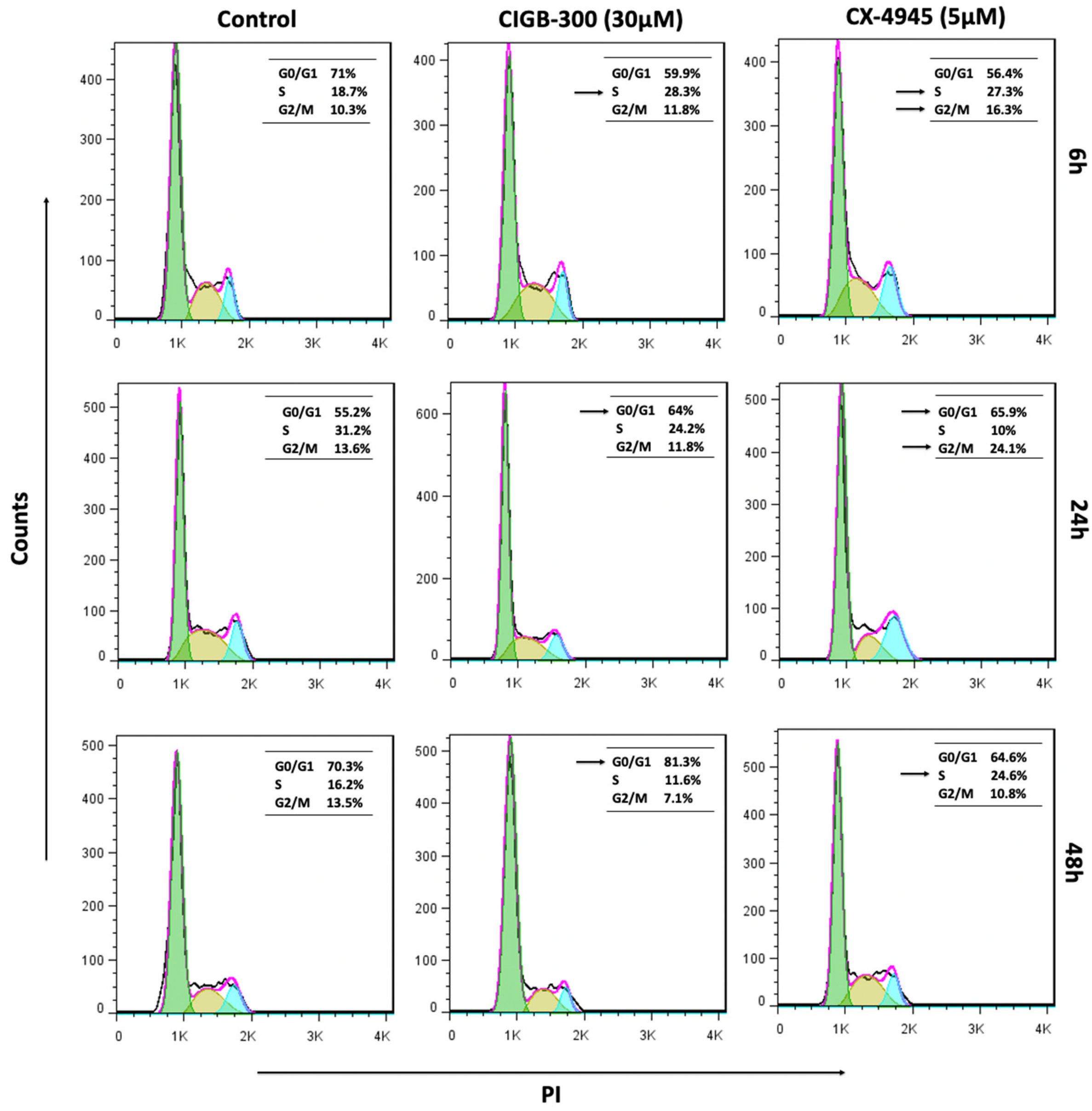

Figure S1. Cell cycle modulation by CIGB-300 and CX-4945 in NCI-H460 6 h, 24 h and 48 h post-treatment. The arrows indicate representative accumulation of cell population in comparison with untreated cells. DNA signals of death cells were excluded from the panel. Dean-Jett-Fox mathematical model was used for the analysis of cells in each phase of cell cycle.
