# Supplementary material for: CIGB-300 Anticancer Peptide Differentially Interacts with CK2 Subunits and Regulates Specific Signaling Mediators in a Highly Sensitive Large Cell Lung Carcinoma Cell Model
Source: Biomedicines. 2022 Dec 25;11(1):43. doi: 10.3390/biomedicines11010043 (PMC9856093; doi:10.3390/biomedicines11010043)
Supplement: Supplementary file 1 [file biomedicines-11-00043-s001.zip › Figure S2.pdf]

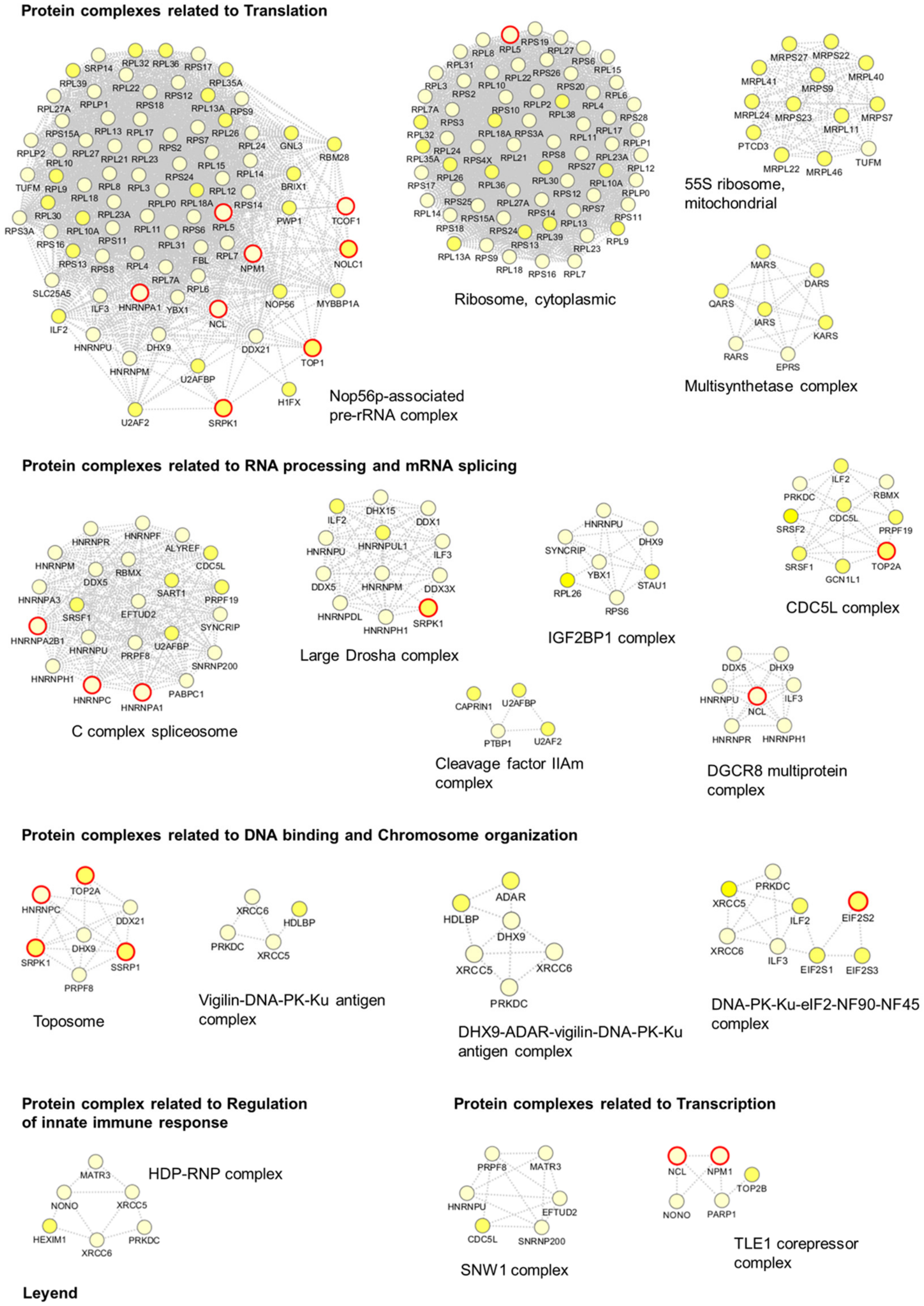

Leyend

- Proteins identified in the CIGB-300 pull-down experiment
- Proteins of the CIGB-300 interactome frequently identified in the CRAPome database

Red border: Well-documented protein kinase CK2 substrates

Figure S2. Protein–protein interaction networks associated with CIGB-300 interacting proteins in NCI-H460 cells. Networks were generated using information gathered from the STRING database and were visualized using Cytoscape. Protein complexes retrieved from the CORUM database are indicated.
