# Supplementary material for: CIGB-300 Anticancer Peptide Differentially Interacts with CK2 Subunits and Regulates Specific Signaling Mediators in a Highly Sensitive Large Cell Lung Carcinoma Cell Model
Source: Biomedicines. 2022 Dec 25;11(1):43. doi: 10.3390/biomedicines11010043 (PMC9856093; doi:10.3390/biomedicines11010043)
Supplement: Supplementary file 1 [file biomedicines-11-00043-s001.zip › Figure S3.pdf]

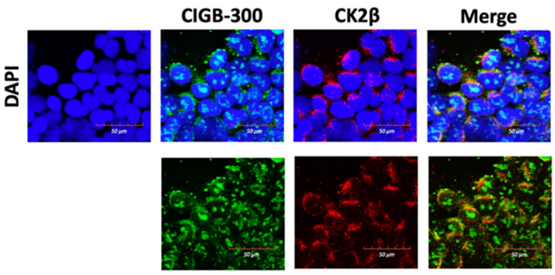

Figure S3. Confocal microscopy in NCI-H460 cells of CIGB-300 and CK2*β*. Blue fluorescence: nuclear DAPI stain. Green fluorescence: CIGB-300-B was incubated at IC_{50} value during 0.5 h and identified with Streptavidin-FITC. Red fluorescence: Corresponding proteins were identified with a primary antibody and then with a secondary antibody conjugated to AlexaFluor 500. Orange fluorescence: Merge of Green/Red channels representing colocalization signals. Scale bars: 50 µm.
